# Diagnostic and Treatment Approaches Involving Transthyretin in Amyloidogenic Diseases

**DOI:** 10.3390/ijms20122982

**Published:** 2019-06-18

**Authors:** Gil Yong Park, Angelo Jamerlan, Kyu Hwan Shim, Seong Soo A. An

**Affiliations:** Department of Bionano Technology, Gachon Medical Research Institute, Gachon University, Seongnam-si 13120, Korea; gilyong8472@gmail.com (G.Y.P.); angelojamerlan@gmail.com (A.J.); smuller0305@gmail.com (K.H.S.)

**Keywords:** transthyretin, mutation, protein aggregation, amyloid, amyloidosis

## Abstract

Transthyretin (TTR) is a thyroid hormone-binding protein which transports thyroxine from the bloodstream to the brain. The structural stability of TTR in tetrameric form is crucial for maintaining its original functions in blood or cerebrospinal fluid (CSF). The altered structure of TTR due to genetic mutations or its deposits due to aggregation could cause several deadly diseases such as cardiomyopathy and neuropathy in autonomic, motor, and sensory systems. The early diagnoses for hereditary amyloid TTR with cardiomyopathy (ATTR-CM) and wild-type amyloid TTR (ATTRwt) amyloidosis, which result from amyloid TTR (ATTR) deposition, are difficult to distinguish due to the close similarities of symptoms. Thus, many researchers investigated the role of ATTR as a biomarker, especially its potential for differential diagnosis due to its varying pathogenic involvement in hereditary ATTR-CM and ATTRwt amyloidosis. As a result, the detection of ATTR became valuable in the diagnosis and determination of the best course of treatment for ATTR amyloidoses. Assessing the extent of ATTR deposition and genetic analysis could help in determining disease progression, and thus survival rate could be improved following the determination of the appropriate course of treatment for the patient. Here, the perspectives of ATTR in various diseases were presented.

## 1. Introduction

Transthyretin (TTR) protein was discovered incidentally from cerebrospinal fluid (CSF) in 1942 and was called prealbumin based on its observed electrophoretic pattern [1]. The name “transthyretin” was formulated from the combination of three words: *trans*port, *thy*roxine and *retin*ol. As the name implied, TTR is a carrier protein of thyroxine and retinol. The gene for TTR is located in chromosome 18 position 12.1 (18q11.2-12.1) or base pair 31,591,766 to 31,599,023 and consists of four exons and five introns [2]. Up to now, over 140 variants [3] have been reported in the TTR gene, where the majority of variants could cause structural abnormalities and implications in disease development. Wild-type TTR still has the tendency to aggregate, and aggregation is not necessarily enhanced despite the presence of amyloidogenic mutations [4]. Only when the mutations are located in β-strands can they increase the chances of destabilizing the native β-structure [4]. Additionally, wild-type amyloid TTR (ATTRwt), where transthyretin deposition occurs sporadically, is still widely misunderstood. The significant involvement of three variants of non-coding regulatory regions and exons in ATTRwt were shown in a cohort of 108 Caucasian males ranging from 59–87 years old and only one (rs72922940) stayed nearly significant following multiple testing corrections [5]. More recently, the prevalence of non-amyloidogenic and amyloidogenic variants of ATTR was estimated from the gnomAD database. Eleven were shown to have effects on function, with c.424G>A as the most prevalent (88% of function-affecting variants) followed by c.148G>A (5%) [6]. The role of TTR as a carrier protein was revealed to involve the reduction of cytotoxicity by blocking the aggregation of other proteins in protein aggregation diseases [7]. TTR was observed to bind to Aβ oligomers *in vitro* with a certain ratio and inhibit the growth and formation of new Aβ aggregates, leading to a reduction in neurotoxicity of Aβ [7]. Two binding sites (EF helix, G strand) of TTR were reported to be a Aβ oligomer sensor and scavenger, respectively [8]. Saturation transfer difference – nuclear magnetic resonance (STD-NMR) spectroscopy together with molecular modeling methods were used to describe the binding of TTR with Aβ(12–28) peptide [9]. Dot blot epitope mapping also further revealed Aβ(18–21) as the main epitope for TTR binding [10]. NMR competition experiments also revealed that Aβ(12–28) bound at the surface of TTR, specifically the RBP binding pocket between the TTR dimers, and a dissociation constant of ~2 μM for Aβ(12–28) [9]. Computational models also showed that the preferred interaction occurred at residues 93–97 of TTR, which formed a hydrophobic patch at the surface pocket of the protein [9]. The association of TTR rare genetic variants with Alzheimer’s disease (AD) was also analyzed in a large cohort of Han Chinese. *In silico* and cell functional studies revealed four potentially pathogenic rare TTR variants in AD: c.-239C>A, which decreased TTR promoter activity; c.200+4A>G, which may influence TTR mRNA inherent splicing; and c.148G>A (p. V50M) and c.332C>T (p.A111V) which may change TTR structure and reduce Aβ binding affinity [11]. Notably, the effective concentrations of TTR were found to be 220–450 ng/mL in healthy adult men and 160–380 ng/mL in adult women [12]. These concentrations served as an indicator in nutritional analysis and became highly significant for obtaining nutritional follow-up data for patients as well as normal individuals.

## 2. Structural Overview: The Role of TTR

**Figure 1:**
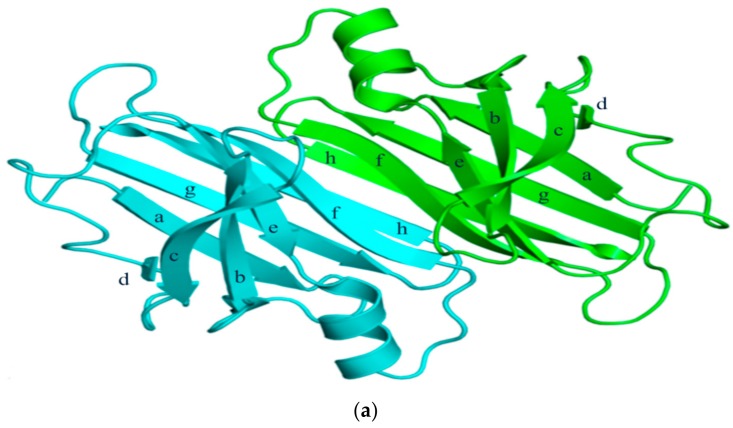

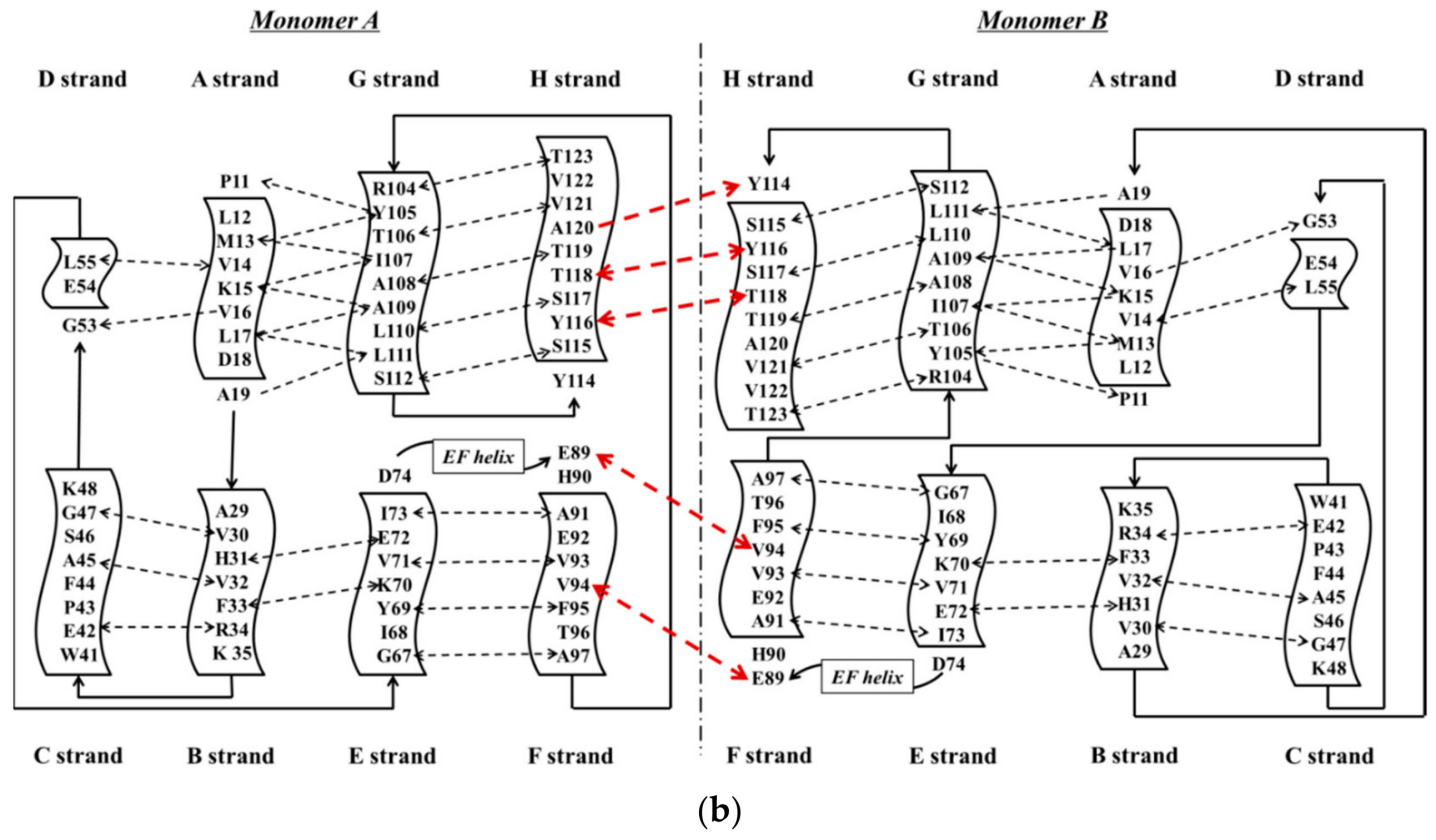
(**a**) 3-Dimensional structure of ATTRwt (PDBe ID code; 1BMZ [18]) in dimeric form; (**b**) Schematic diagram of hydrogen bonds in transthyretin and monomer–monomer’s hydrogen bonds. Arrowhead indicates acceptor, the tail indicates donor. In acidic condition, hydrogen bonds will be broken and destabilized.

TTR has a molecular weight of 14 kDa in monomeric form and exists in tetrameric form (55 kDa) in plasma [13]. It is synthesized in the liver and choroid plexus as a homo-tetramer structure with a dimer of dimers quaternary structure. The amino acid sequence from 1 to 20 is the signal peptide, and the final protein structure is expressed from amino acids 21 to 147. Hence, each monomer consists of 127 amino acids with one alpha helix, called EF helix, and eight beta strands (Figure 1a) [14,15]. Hydrogen bonds primarily kept the monomer and dimer structures intact. The pairs of hydrogen bonds in D strands of the monomers contributed to structural stability by forming an internal convex-shaped channel (Figure 1b) [16]. The two dimers then gathered to form a homo-tetramer through additional hydrogen bonds, finalizing the assembly of the carrier protein [17].

**Figure 2:**
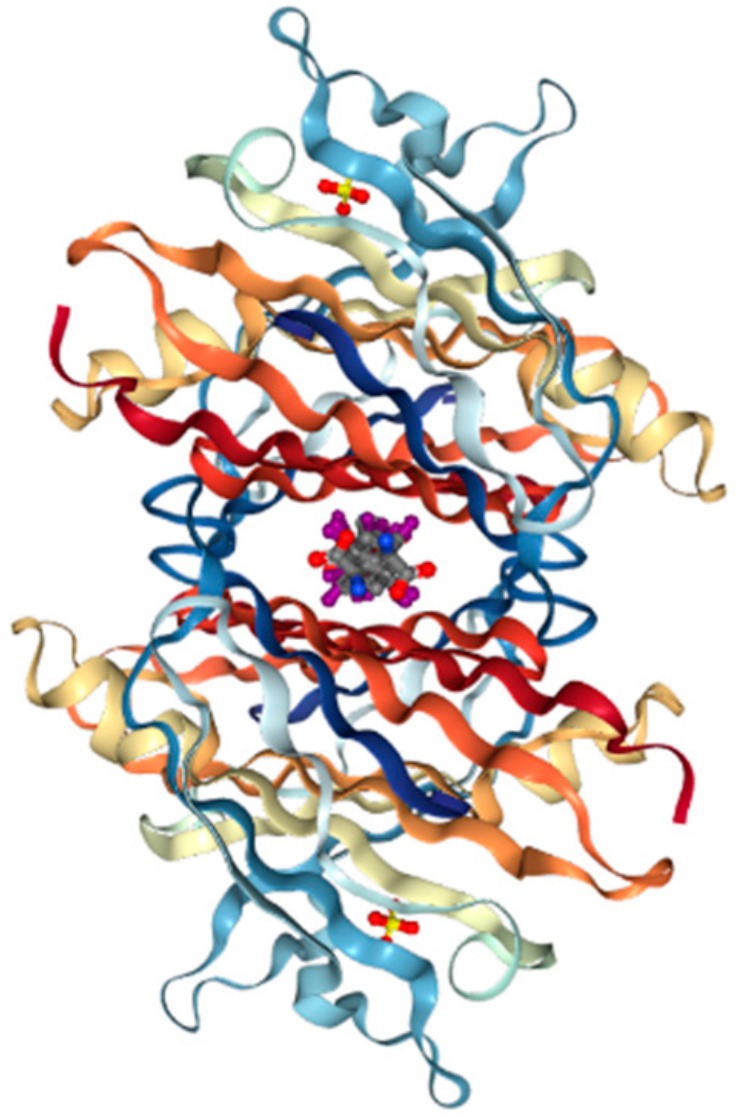
Transthyretin—T4 hormone binding structure. (PDB ID code; 2ROX [21]).

Production of TTR by the liver or choroid plexus is followed by the translocation of the protein into the blood or CSF. The role of homo-tetrameric TTR is to transport T4 hormone and retinol. More than 99% of human T4 hormones are transported by carrier proteins, such as albumin, thyroxine-binding globulin, and TTR, and these same proteins are responsible for 15% of T4 hormone transports in circulation [19]. Homo-tetrameric TTR has two binding sites at a central channel called T4 pocket and is formed by side chain interactions [16]. Earlier reports of T4 binding studies predicted only one binding site, but investigation using a fluorescent probe, 8-anilinonapthalene-1-sulfonate, revealed two binding sites at opposite ends. Despite TTR having two T4 hormone-binding sites, the T4 hormone would preferentially bind to only one of the two sites. This preferential binding is influenced by relatively stronger binding affinity of one binding site and negative cooperativity, as well as structural changes of TTR, which can significantly reduce affinity at the other binding site [20]. Finally, the formation of hydrogen bonds between T4 hormone and Lys15 at the A strand/Glu54 at the D strand reinforced the hormone at the center of the tetramer (Figure 2) [21].

On the other hand, vitamin A (retinol) binds to TTR through a mediating protein, called retinol-binding protein (RBP) instead of the T4 binding protein. RBP (20 kDa) is synthesized by hepatocytes in the liver and consists of one alpha helix and eight anti-parallel beta-barrels [22]. Two RBPs bind and surround the TTR tetramer from both sides, but the limited concentration of RBP would result in a 1:1 molar ratio in plasma [23]. The amino acids of TTR interact with the following amino acids of RBP: Leu35, Trp67, Lys89, Trp91, Ser95, Phe96, Leu97 and Lys99 [24]. Retinol could not escape renal filtration through the urine once bound to the complex, TTR-RBP, thereby preserving retinol concentration as well as that of RBP [25,26]. The stability of the TTR-RBP-retinol complex is reduced when retinol is removed [27].

## 3. ATTR Causative Diseases

**Figure 3:**
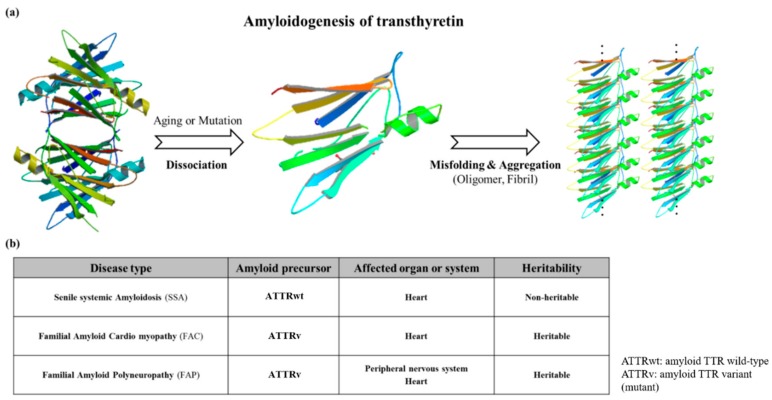
(**a**) Schematic diagram showing the amyloidogenesis of transthyretin. Transthyretin amyloidosis requires tetramer dissociation to monomer. (Transthyretin tetramer—PDB ID code; 4PVL83, Transthyretin monomer—PDB ID code; 3I9A84, Fibrils structure is made by ATTRv monomer without calculation); (**b**) Types of transthyretin amyloidoses.

Mutations which are present in the TTR core structure reduce the stability of the protein. As a result, the TTR tetramer could separate into dimeric and monomeric forms, and these monomers coalesce by forming amyloid TTR (ATTR) aggregates (Figure 3) [28]. This aggregation, called amyloidogenesis, progresses to plaque formation, later depositing into tissues and neurons and inducing cytotoxicity. The different amyloidogenic diseases that present as a result were initially termed as the following: senile systemic amyloidosis (SSA), familial amyloid cardiomyopathy (FAC), and familial amyloid polyneuropathy (FAP) [28,29]. SSA, FAC, and FAP were reported to share the same accumulation of ATTR, but the types of ATTR found were different for each disease. In the case of SSA, aggregated constituents were composed mainly of large quantities of deposited wild-type ATTR (ATTRwt) in myocardia [30,31]. Updates in nomenclature were recently recommended by the International Society of Amyloidosis (ISA) to modify the terms SSA, FAC, and FAP to more exact definitions due to overlap in clinical presentation [32]. SSA was renamed as wild-type amyloid TTR amyloidosis (ATTRwt amyloidosis). It usually occurs in 25% of the elderly population over 80 with higher frequency in males at 25~50: 1 ratio [33,34]. Large amyloid deposits in the myocardia could cause congestive heart failure [35] and ventricular hypertrophy when echocardiography showed thickened ventricular walls along with low QRS voltages from electrocardiography (ECG) [36]. Common observable symptoms are fatigue, edema, shortness of breath, chest pain, and angina, and the severity of symptoms from these diseases may vary depending on the degree of ATTRwt deposits [37,38,39].

**Figure 4:**
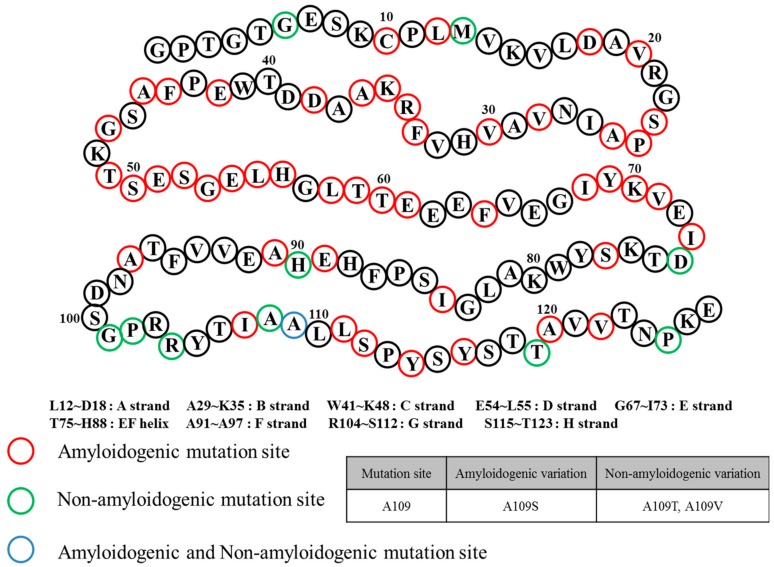
Transthyretin mutation sites and relation to amyloidosis.

FAC/FAP belong to the same pathological spectrum and differ from ATTRwt amyloidosis mainly due to ATTR mutations. In this case, ATTRv (amyloid TTR variant) refers to the mutant form of ATTR, and FAC/FAP was renamed to ATTR appended with the specific mutation that caused it (i.e., ATTRV30M) and the accompanying symptom (i.e. ATTR with cardiomyopathy) [32]. Depending on the mutations, the hydrophobic interaction of dimer–dimer formation would vary, resulting in the instability and breakdown of the tetramers. As a result, the tendency to produce the amyloid form is enhanced and favors the development of the disease [40]. FAP was formerly categorized into four types: FAP-I, FAP-II, FAP-III and FAP-IV, and the pathogenicity of FAP and FAC could overlap [41,42,43]. FAP-I has since been renamed as ATTRV30M amyloidosis and is also known as the Portuguese–Swedish–Japanese type, and haplotype comparison among foci suggested common Portuguese origins among Japanese, Spanish, and Brazilian patients, while Swedish patients had entirely different haplotypic origins [44,45]. ATTRV30M amyloidosis resulted in a wide range of symptoms such as sensory-autonomic, gastrointestinal and cardiac disturbances, impotence, and cardiomyopathy; as well as renal insufficiency in later stages of the disease [46,47]. A study on 15 Swedish families with ATTRV30M determined through Western blot showed the type of amyloid fibril composition remained the same despite the differences of the disease age of onset. Only one family showed different types of amyloid fibril composition on two brothers with similar ages of disease onset [48]. Despite the endemicity of the early onset form of ATTRV30M in some Portuguese, Japanese, and Swedish populations, the late onset form was shown to be more prevalent in non-endemic areas of Japan and Cyprus than previously thought, and much is still not known about its causative factors [49,50]. A similar comparative study on early and late onset ATTRV30M Portuguese patients showed an equal sex and geographical distribution of both disease groups [51]. Notably, the early onset group showed more frequent family history compared to the late onset group. The late onset group, however, presented more organ involvement and frequent neuropathic pain [51]. Another study on Brazilian patients showed more severe neurologic and cardiac impairments of the late onset form of ATTRV30M amyloidosis as well as its high frequency of misdiagnosis, further illustrating distinct clinical presentations in spite of sharing the same mutation [52]. Interestingly, surface-enhanced laser desorption/ionization time-of-flight mass spectrometry (SELDI-TOF MS) showed ATTRv deposits in early onset cardiac amyloid samples while more than half were ATTRwt in late onset samples, suggesting the possible significant role of ATTRwt in late onset amyloidosis [53]. ATTRI84S amyloidosis (formerly FAP-II) was called Indiana/Swiss or Maryland/German type [54]. Symptoms of ATTRI84S amyloidosis were similar to ATTRV30M amyloidosis, but additional sensorimotor polyneuropathy could appear late in the disease [55]. In addition, amyloid deposits could be found in the eye, thyroid, adrenal glands, and blood vessels, leading to additional side effects from both diseases [56,57,58,59]. Even though ATTRv amyloidosis was like ATTRwt amyloidosis in terms of disease pathomechanism, the expression of ATTRv outside cardiac tissue may lead to amyloidosis resulting in hereditary ATTR cardiomyopathy (ATTR-CM). This was described in a Danish family carrying ATTRMet^111^ expressed in plasma that led to hereditary ATTR-CM (previously known as familial amyloid cardiomyopathy or FAC), whereas unafflicted members were seronegative [60]. Patients beyond the age of 60 were reported to have hereditary ATTR-CM, and ATTRV122I and ATTRT60A could be responsible. ATTRV122I was most commonly found in 4% of African-Americans, followed by ATTRT60A in patients from United Kingdom and Ireland [61,62,63]. The role of the non-coding variation of ATTRV122I was assessed in 4,361 unrelated African-Americans. The study showed that this allele increased 6.8-fold the risk of having ten or more outpatient surgeries. Additionally, men had a 15.2-fold higher risk of having ten or more outpatient surgeries. This non-coding variation seemed to accelerate the negative consequences associated with ATTRV122I amyloidosis [64]. Amyloid deposits in cardiac tissues may cause a thickening of cardiac walls leading to congestive heart failure and atrial arrhythmias, and result in cardiac arrest and death [59,65]. Carpal tunnel syndrome may manifest in the early phase of ATTRv amyloidosis and may require a great deal of attention and not simply dismissed as an unrelated symptom [66]. More than 140 ATTRv mutations have been identified [3] along with non-amyloidogenic mutations (Figure 4), which may not produce amyloid, but were reported to cause functional abnormalities *in vivo* [67,68]. Among non-amyloidogenic mutations, few were revealed to have a high affinity for thyroxine [67], and rarely, alanine mutations, particularly A109T and A109V, which were described in a family with dominantly-inherited euthyroid hyperthyroxinemia [69].

## 4. General Diagnostic Workflow for ATTR Causative Diseases

Upon initial detection of the clinical symptoms mentioned above, additional diagnoses are still required to discern ATTR disease types for prompt treatment. ECG, magnetic resonance imaging (MRI), echocardiography, tissue biopsy, and genetic analysis could be performed for wild-type and hereditary ATTR amyloidoses. ECG is prioritized if the detected symptoms are primarily observed in the heart. In recent years, many kinds of ECG analysis were reported to lead to better initial diagnosis. As an example, left bundle branch block can differentiate ATTRwt amyloidosis from primary light chain amyloidosis (AL) as this ECG pattern can be observed in 40% of ATTRwt patients but is rare (4%) in AL [70]. Notably, low QRS voltages were observed in 60% of AL patients but were not as frequent in ATTRwt patients (40%) [70,71]. An abnormal ECG result would call for MRI using gadolinium enhancement by visualizing amyloid deposition in cardiac tissue for a more accurate diagnosis [72]. Echocardiography was used for diagnosing hypoplasia in the left and right ventricles of neonates [73] and has also been considered as an invaluable tool in providing real time and rapid evaluation of ventricular function in neonates and children with suspected ventricular anomalies [74]. This is largely in part due to its noninvasive nature and absence of side effects during right ventricular evaluation [75] which consists of assessment of ventricular atrophy based on ventricular muscle thickness [76]. Echocardiography also proved to be equally valuable in the assessment of left ventricular function through the measurement of different parametric velocities of the ventricular wall [77]. More recently, global longitudinal strain (GLS) measurement through speckle-tracking analysis of 2D-echocardiography was demonstrated to be a feasible non-invasive and more accurate alternative to the more traditional left ventricular ejection fraction (LVEF) [78]. Tissue biopsy could more accurately differentiate whether the disease was due to wild-type or variant-type ATTR deposits compared to simple visualization. If ATTR amyloidosis was suspected following MRI or 2D-echocardiography, tissue should be collected from the appropriate site depending on the patient’s condition, such as the heart tissue and sural nerve [79]. Each tissue was analyzed for ATTR types through amyloid fibril analysis using Congo red, immunohistochemistry, and mass spectrometry [80,81,82]. Despite its accuracy, acquiring tissue samples internally through biopsy is an invasive procedure when compared to ECG and 2D-echo, thus other more easily accessible organ sources were considered. Not too recently, skin biopsy was shown to be a promising alternative to the otherwise more invasive method of acquiring internal tissues from the heart or sural nerve in ATTRv (FAP) diagnosis [83]. Intraepidermal, sweat gland, and pilomotor nerve fiber densities were measured and compared in ATTRv patients, asymptomatic ATTRv carriers, healthy controls, diabetic neuropathy disease controls, and AL patients [83]. Immunohistochemistry revealed decreased fiber densities from all three tissue sources in ATTRv patients compared to normal controls, while ATTRv carriers showed intermediate reductions. The sensitivity and specificity for ATTRv diagnosis through detecting amyloid in skin was calculated to be 70% and 100%, respectively [83]. Additional genetic analyses using blood can be done to determine the type of disease. ATTR variants were identified by PCR-based full sequence analysis with 99% accuracy [84,85]. From these methods, patients with ATTRV112I were diagnosed as ATTR-CM (FAC at the time) [63], and the ATTRV30M variant could suggest amyloid deposition in the cardiac muscle or nerve [86]. If no mutation was found, the patient would be diagnosed with ATTRwt amyloidosis [30]. Thus, amyloidosis could be diagnosed by combining the complementary results of several diagnostic methods, and if the disease was suspected as one with hereditary origin, continuous follow-up observation was required.

## 5. Role of ATTR as Biomarker for Amyloidosis

Due to the diverse roles and interactions of TTR, its status as a biomarker received much attention, and, in fact, an increasing number of reports strongly suggested ATTR as a potential leading biomarker for ATTRwt and ATTRv amyloidoses and euthyroid hyperthyroxinemia [30,41,42,43,63,69]. A recent report suggested that ATTR in CSF was upregulated in patients with AD or frontotemporal dementia (FTD) involving mutant C9orf72 in comparison to disease control groups and control groups without neurological diseases and without mutant C9orf72 [87]. This was expanded upon by an extensive review on the role of TTR expression on oxidative stress [88]. Emphasis was given on the direct connection of AD and TTR expression as a normal physiological reaction to alleviate oxidative stress due to the deposition of Aβ. This was supported by the increased TTR expression in patients with neurodegenerative disorders where oxidative stress is a key contributor to pathophysiology [89]. Peptide-based probes were also designed to selectively detect ATTR in plasma of hereditary ATTR amyloidosis patients [90]. The probes showed decreased amounts of ATTR in healthy controls as well as in asymptomatic carriers of mutations associated with the disease [90]. Interestingly, the probe also revealed a circulating TTR fragment that disappeared following tafamidis treatment in a subset of TTR amyloid neuropathy patients. Proteomic analysis of this peptide led to suggest that TTR cleavage happens between TTR tetramer dissociation and deposition into oligomers [90]. The irregularities in endoproteolysis implied unique proteotoxicity mechanisms, which may help explain differences in disease onset for ATTRV30M patients and the accelerated progression of hereditary ATTR amyloidosis in males compared to females [90].

## 6. Treatment Methods of TTR Amyloidosis

The treatment of ATTR amyloidosis could be divided into the following approaches. These prevent the onset of ATTR aggregation or eliminate protein structural abnormalities depending on the type of disease. For patients with ATTR amyloidosis with polyneuropathy, orthotopic liver transplantation (OLT) was considered to be the primary treatment [91]. OLT was shown to reduce ATTR in blood, gradually relieving the resulting neuropathy [92]. However, OLT could not always remove completely ATTR depositions from malfunctioning organs or tissues, thus requiring individual organ transplantation or antibody therapy as an alternative approach [93,94]. Due to many practical difficulties of transplantation, one of the treatment options could be symptom management, which can be applied to both ATTRwt and ATTRv amyloidoses [95,96]. Three major types of targeted drug therapy involved blocking the production of ATTR, increasing the structural stability of the tetramer, and removing the deposited fibrils. Recent studies suggested the possibility of antisense oligonucleotides (ASO) and small interfering RNA (siRNA) as genetic therapeutic agents for blocking ATTR expression. ASOs and siRNAs could cleave mRNA prior to protein synthesis and inhibit ATTR production. Since these could not be transported alone *in vivo*, biodegradable carriers were also considered for intravenous infusion [97,98,99,100]. These methods of gene silencing inhibited up to 80% of expressed ATTRv and ATTRwt in liver and choroid plexus. Recently, Inotersen/Tegsedi^®^ (Akcea Therapeutics), a second generation ASO specific for inhibiting the production of ATTR by the liver, completed the Phase III NEURO-TTR study and was shown to be effective at stabilizing and improving the quality of life evaluated by the Norfolk Quality of Life—Diabetic Neuropathy (QOL-DN) questionnaire [101]. The first siRNA-based drug in history to be recently approved by the US FDA for the treatment of hereditary transthyretin amyloidosis was patisiran, which directly binds to mRNA, preventing the expression of both ATTRwt and ATTRv [102,103]. Patisiran showed consistent slowing and inhibition of neuropathy in Phase II and III, and long term studies [103]. A review by Yang discussed the chemical properties, mechanism of action, pharmacokinetics, clinical safety, and efficacy of patisiran [104]. It was concluded that patisiran was generally safe and well tolerated; significantly improved neuropathy with minor issues such as deficiency in vitamin A levels since TTR is required for retinol transport; therefore, regular vitamin A supplementation may be required [104]. On the other hand, tafamidis, diflunisal and flufenamic acid bind to the T4 binding sites of the TTR tetramer and increase their own structural stability, thereby inhibiting the aggregation of amyloid plaques. Hence, many trials and prototypes were applied. Tafamidis failed to gain the approval of the US FDA back in September 2010, but the European Union later approved it on November 2011. The efficacy of tafamidis was recently evaluated in a multicenter, international, double-blind, and placebo-controlled phase 3 trial consisting of 441 ATTR-CM patients which received 2:1:2 treatment ratio consisting of 80 mg tafamidis, 20 mg tafamidis, or placebo in a course of 30 months [105]. Primary analysis showed all-cause mortality and rates of cardiovascular hospitalizations were lower in 264 tafamidis-treated patients compared to 174 placebo controls (*p* < 0.001) as well as reduced decline in functional capacity and quality of life [105]. There were other attempts to develop drugs to ameliorate fibril deposition [106,107,108,109]. An antibody (misTTR) that targeted the 89–97 residues of TTR was developed and nanomolar concentrations of misTTR inhibited fibrillogenesis of micromolar concentrations of ATTR [109]. This approach successfully demonstrated that fibrillogenesis can be ameliorated by alternatively targeting ATTR intermediates over native state stabilization. Notably, substoichiometric concentrations were key to achieving effective inhibition of ATTR fibrillogenesis [109]. The representative example was the doxycycline-taurodeoxycholic acid complex (doxycycline-TUDCA). When human F30 transgenic mice expressing ATTRV30M were treated daily with 8 mg of doxycycline-TUDCA per kilogram body weight for 15 days, the amount of amyloid observed was decreased. Interestingly, the combined regimen of doxycycline and taurodeoxycholic acid demonstrated increased potency in the early stage of treatment [110]. According to a paper from a phase II clinical trial of doxycycline-TUDCA involving 20 patients, the toxicity was acceptable during follow-ups that lasted for one year. Seventeen patients were diagnosed with hereditary ATTR amyloidosis, two patients with ATTRwt amyloidosis and one patient with domino recipient. Any disease progression related to cardio and neuropathy was not shown [111].

## 7. Conclusion and Discussion

**Figure 5:**
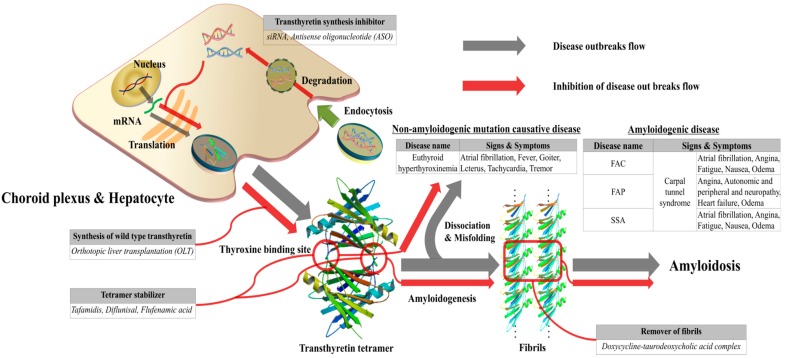
Schematic diagram of transthyretin disease and treatment strategies. (Transthyretin tetramer—PDB ID code; 4PVL83, Transthyretin monomer—PDB ID code; 3I9A84, Fibrils structure is made by muTTR monomer without calculation).

Monomeric TTR (127 a.a. after the truncation of signal sequence from 147 a.a.) is expressed autosomally and dominantly from hepatocytes or choroid plexus (Figure 5). Two TTR monomers form a dimer, and the formed dimers subsequently form a tetramer. The main role of TTR would be to carry thyroxine and retinol *in vivo*. In addition, TTR could inhibit the production and growth of Aβ oligomers, alleviating the development of neurotoxicity due to Aβ aggregation. Transthyretin, bearing close similarity to Aβ in terms of amyloidosis, could be misfolded into its protein amyloid aggregates and cause several diseases. Depending on the ATTR aggregates, the type of diseases could vary and may be classified as either ATTRwt or ATTRv amyloidosis. ATTRv could be further classified into ATTRV30M amyloidosis (Portuguese-Swedish-Japanese type) or ATTRI84S amyloidosis (Indiana/Swiss or Maryland/German type). In addition, the formation of amyloid deposits aggregated from ATTRwt can lead to ATTRwt amyloidosis. However, euthyroid hyperthyroxinemia is not caused by amyloid-derived aggregates, but by the non-amyloidogenic mutations. Differential diagnoses could be made by employing various methods with blood and tissue samples. ECG, MRI, and echocardiography could also provide additional information in the final diagnosis. Despite these different causes, it is possible to treat ATTR amyloidosis through common approaches because the disease is usually caused by the deposition of ATTR due to structural instability. OLT was considered the treatment of choice and has been widely demonstrated to be successful. However, due to underlying indirect causes of OLT, especially its invasive nature, it could not be performed on all patients. As an alternative approach, drug stabilizers were proposed to prevent dissociation of TTR tetramer caused by structural abnormalities and maintain its native structure. In this case, a phase II study utilizing this approach to remove the fibrils was reported to show significant results. Several other studies were conducted to obtain similar results acquired through OLT, including gene therapy. Treatments with ASO and siRNA were suggested as emerging and promising approaches, and two, in the form of Inotersen/Tegsedi^®^ and patisiran, already presented promising results in significantly improving neuropathy and quality of life of ATTRv patients. This resulted in patisiran being the first siRNA-based therapy to be approved recently by the USA FDA. More diverse investigations on novel treatment strategies and other genetic causalities are still needed to understand the pathological role of ATTR in amyloidoses. Such studies may uncover hidden pathways in disease pathogenesis and may significantly improve methods for differential diagnosis and treatment.
